# The Expanding Role of the Oncology Pharmacist

**DOI:** 10.3390/pharmacy8030130

**Published:** 2020-07-25

**Authors:** Lisa M. Holle, Eve M. Segal, Kate D. Jeffers

**Affiliations:** 1 School of Pharmacy, University of Connecticut, 69 North Eagleville Road, Storrs, CT 06268, USA; 2 Pharmacy Department, Seattle Cancer Care Alliance, Seattle, WA 98109, USA; segaleve@seattlecca.org; 3 Pharmacy, UCHealth Southern Colorado Region, Colorado Springs, CO 80909, USA; Kate.Jeffers@uchealth.org

**Keywords:** hematology/oncology, oncology, pharmacist, pharmacy

## Abstract

Although oncology pharmacists have been involved in the care of cancer patients for over 50 years, the role of the oncology pharmacist continues to expand. Initially, pharmacists were primarily based within either an inpatient or outpatient pharmacy setting and their work focused on providing the necessary safety checks to dispense cancer-related medications. With technology freeing up pharmacists from dispensing functions and advanced training in direct patient care (e.g., oncology residency, oncology fellowship), the oncology pharmacist was able to provide direct patient care at the bedside or within the clinic where treatment decisions are made by the healthcare team. In fact, they have become integral members of the healthcare team. This Issue describes several expanding roles of oncology pharmacists in stem cell transplant, hematology, gastrointestinal oncology, and precision genomics, as well as oncology pharmacists preventing a reduction in cancer patient visits as the oncology physician shortage occurs. Oncology pharmacists are an integral part of the cancer care team; their value has been documented in several studies, and is highlighted in this Issue. We encourage the profession to continue to document their value so that one day each patient can have an oncology pharmacist as part of their cancer care team.

Although oncology pharmacists have been involved in the care of cancer patients for over 50 years, the role of the oncology pharmacist continues to expand [1,2]. Initially, pharmacists were primarily based within either an inpatient or outpatient pharmacy setting and their work focused on providing the necessary safety checks to dispense cancer-related medications [2]. With technology freeing up pharmacists from dispensing functions and advanced training in direct patient care (e.g., oncology residency, oncology fellowship), the oncology pharmacist was able to provide direct patient care at the bedside or within the clinic where treatment decisions are made by the healthcare team. In fact, they have become integral members of the healthcare team [3]. With their training and expertise, they can assist with providing evidence-based care to the patient with cancer. Some of their daily responsibilities include educating patients and caregivers about their therapies, improving medication adherence, and educating other members of the healthcare team on medications used in cancer care. Because of their clinical knowledge, literature evaluation skills, and understanding of the complexity of cancer care, they often are involved in the development of guidelines, policies, standards, and clinical pathways both at the institutional level but also at regional, national, and international levels.

Common roles for oncology pharmacists have included inpatient pharmacist, ambulatory pharmacist, infusion center or decentralized pharmacist, specialty pharmacist, practice manager, and investigational drug pharmacist [4]. An inpatient oncology pharmacist typically is responsible for the pharmacotherapy management of patients with cancer while they are hospitalized. They work closely with nursing staff to coordinate chemotherapy administration and provide patient and staff education. The ambulatory oncology pharmacist is responsible for medication therapy management for patients with cancer from diagnosis throughout survivorship. They may work under a collaborative practice agreement to prescribe supportive care medications or adjust anticancer medications as well as provide patient education. Infusion center or decentralized oncology pharmacists are involved with the sterile compounding of anticancer treatments and related supportive care medications, and may support some of the direct patient care functions of the ambulatory oncology pharmacists. Specialty oncology pharmacists are involved with distribution and dispensing of oral anticancer treatments. They may work at a specialty pharmacy that is located within the Cancer Center or at an external site, and provide patient education, perform adherence monitoring, and assess patient safety. Oncology practice managers oversee oncology pharmacists, and are often involved in facilitating the ability of the oncology pharmacy team to provide safe and effective care, manage fiscal and personnel resources, develop policies and procedures, and develop strategic quality initiatives. They may also have clinical responsibilities at some institutions. Investigational drug pharmacists are responsible for coordinating the processes related to oncology investigational drug studies in accordance with legal, professional, institutional, and sponsor requirements. Their core responsibility is to ensure the patient’s access to investigational drugs and serve as the medication expert to all involved in these research studies. Although these are the most common roles of oncology pharmacists, many other roles exist. These include academia, medical communications, population health management, informatics, companies associated with providing health or pharmacy benefits, manufacturers, wholesalers, and regulatory agencies.

The value of the oncology pharmacist in the care of patients with cancer and within the oncology care team has been documented in several studies [5]. Oncology pharmacists have demonstrated their value in providing clinical care that has directly impacted patient outcomes, supportive care management, laboratory monitoring, and increased documentation in the electronic medical record [6,7,8,9,10,11,12,13,14,15,16,17,18,19,20,21,22,23,24]. Patient education, a common component of most oncology pharmacist positions, has been shown to be associated with high rates of patient satisfaction, improved learning outcomes, and leads to increased medication adherence and disease-based outcomes [6,17,19,25,26,27,28,29,30]. Oncology pharmacists have become important members of informatics teams, and their integral role has been shown to be associated with increased rates of medication error identification [9,30,31]. Pharmacist-driven cost-savings have been reported in numerous studies, including those using pharmacists to manage oral anticancer therapies, by providing effective inpatient and ambulatory care and implementing quality improvement programs [18,29,31,32,33,34,35,36,37]. Finally, pharmacists can decrease physician and advanced practice provider (APP) time by developing independent practice models and performing tasks typically completed by physicians [7,31,33,36].

A shortage of oncology physicians exists globally, and is expected to continue for several years [38]. Oncology pharmacists are well poised to assist with preventing a reduction in cancer patient visits. In fact, in this Issue of *Pharmacy*, Knapp and Ignoffo have shown that board certified oncology pharmacists (BCOPs) could contribute to outpatient oncology patient visits, thereby preventing the potential of a shortfall of providers available to care for patients with cancer [39]. With the overlap of clinical activities of BCOPs and APPs, BCOPs may also be able to assist with preventing burnout that often occurs in the oncology healthcare team. Thus, this study demonstrates that improved efficiency and effectiveness of the oncology healthcare team can occur with the expanding role of the oncology pharmacist on care teams.

This journal explores four cases of pharmacists integrating themselves in different care teams, such as stem cell transplant, hematology, gastrointestinal oncology, and precision genomics [40,41,42,43]. Some of these clinic activities allow pharmacists to become physician extenders. For example, pharmacogenomic pharmacists are involved in interpreting genomic sequencing reports and subsequently making treatment recommendations [40], whereas other clinics allow pharmacists to practice at the top of their license. In the review by Chen and colleagues [41], pharmacists involved in the management of patients with leukemia can perform independent patient visits and manage patient toxicities related to oral chemotherapy. Furthermore, Darling and colleagues [42] demonstrated that by integrating a pharmacist into the oncology care team, patients’ understanding of their medication improves, as well as their satisfaction. Lastly, a report by Dr. Clemmons [43] demonstrates that having a pharmacist in stem cell transplant can improve care using collaborative practice agreements. 

Oncology pharmacists are an integral part of the cancer care team. They are critical in expanding and enhancing patient care. Their value has been documented in several studies and is highlighted in this Issue. We encourage the profession to continue to document their value so that one day each patient can have an oncology pharmacist as part of their cancer care team.
